# Psychological, social, and neurobiological correlates of non-suicidal self-injury and suicidal behavior in adolescents: a narrative review

**DOI:** 10.3389/fpsyt.2026.1867397

**Published:** 2026-08-05

**Authors:** Mengyao Zhang, Hongli Sun

**Affiliations:** 1 School of Clinical Medicine, Shandong Second Medical University, Weifang, Shandong, China; 2 Sixth Department of Psychiatry, Weifang Mental Health Center, Weifang, Shandong, China

**Keywords:** adolescents, narrative review, non-suicidal self-injury, suicidal behavior, suicidal ideation, suicide attempt

## Abstract

**Background:**

Non-suicidal self-injury (NSSI) is highly prevalent during adolescence and is consistently associated with suicidal ideation (SI) and suicide attempts (SA). Although numerous psychological, social, and neurobiological correlates of the NSSI–suicidal behavior relationship have been investigated, current evidence remains fragmented, and the extent to which these correlates are specific to NSSI rather than reflecting broader psychopathology remains unclear. This narrative review aimed to synthesize current evidence on the psychological, social, and neurobiological correlates of the NSSI–suicidal behavior relationship in adolescents, while highlighting methodological limitations and future research priorities.

**Methods:**

A structured literature search was conducted in PubMed, Web of Science, PsycINFO, and China National Knowledge Infrastructure (CNKI) to identify relevant studies published between January 2010 and June 2025. Studies involving adolescent populations that examined NSSI, suicidal ideation, suicide attempts, or their psychological, social, or neurobiological correlates were included. Consistent with the methodology of a narrative review, studies were selected based on conceptual relevance without formal risk-of-bias assessment.

**Results:**

Current evidence consistently identifies NSSI as a significant risk marker for suicidal ideation and suicide attempts, although the predominantly observational nature of the literature precludes causal inference. Psychological correlates include cognitive flexibility and acquired capability for suicide; social correlates include family functioning, peer influences, and sex differences; and neurobiological studies implicate alterations in the hypothalamic–pituitary–thyroid (HPT) axis, hypothalamic–pituitary–adrenal (HPA) axis, and endogenous opioid system. Dialectical behavior therapy (DBT) demonstrates the strongest evidence for reducing both NSSI and suicidal behaviors in adolescents. However, interpretation of the available evidence is limited by cross-sectional designs, inconsistent definitions and measurements, biological heterogeneity, and inadequate control for psychiatric comorbidity.

**Conclusion:**

The relationship between NSSI and suicidal behavior in adolescents is shaped by interacting psychological, social, and neurobiological factors rather than a single mechanism. Current evidence also highlights the importance of distinguishing factors associated with the emergence of suicidal ideation from those related to the transition from ideation to suicide attempts. Future prospective, multi-method studies integrating these domains while accounting for psychiatric comorbidity are needed to clarify developmental pathways and improve risk assessment and intervention strategies.

## 1 Introduction

Non-suicidal self-injury (NSSI) is defined as the direct and deliberate destruction of one’s own body tissue without suicidal intent (1). Common forms include cutting, scratching, burning, and hitting (2). In the Diagnostic and Statistical Manual of Mental Disorders, Fifth Edition (DSM-5), Nonsuicidal Self-Injury Disorder is included in Section III as a condition for further study, rather than as a fully established independent diagnosis (3).

Several related concepts should be distinguished at the outset. NSSI refers to self-injury without suicidal intent. Self-harm is a broader term that may or may not include suicidal intent, depending on the definition employed in individual studies. Suicidal ideation (SI) refers to thoughts about ending one’s life, ranging from passive wishes of death to active planning. A suicide attempt (SA) is a potentially self-injurious behavior with at least some intent to die. Suicidal behavior (SB) is an umbrella term encompassing suicidal ideation, suicide attempts, and suicide death.

Globally, the lifetime prevalence of NSSI among adolescents is estimated at 22.0%, with substantial regional variation (4, 5). According to the World Health Organization, suicide is the third leading cause of death among adolescents worldwide (6). NSSI, SI, and SA are important risk markers for future suicidal behavior and constitute a serious public health problem (4, 5, 7, 8).

NSSI imposes substantial emotional and economic burdens on affected adolescents, their families, healthcare systems, and communities. Parents and caregivers of adolescents with NSSI may experience elevated psychological distress, including depression, anxiety, shame, and guilt, with some reporting feelings of helplessness and social isolation (9, 10). Notably, parents of adolescents with self-injurious thoughts and behaviors report significantly higher stress and distress compared to parents in control groups, which may compromise their capacity to support their child’s recovery (11). The direct medical costs associated with self-harm—including emergency department visits, inpatient hospitalizations, and outpatient mental health services—represent a substantial financial burden on healthcare systems (12). A study in the United States estimated that the annual economic cost of nonfatal self-harm injuries among children and young adults (aged 10–44 years) averaged approximately $19 billion, encompassing medical treatment, lost productivity, and other indirect costs (12). While comparable comprehensive cost data for other countries are limited, the high global prevalence of NSSI among adolescents suggests that the economic burden is substantial worldwide. An analysis of Global Burden of Disease 2019 data found that self-harm accounted for a considerable proportion of disability-adjusted life-years among young people in Europe, with higher burdens observed in countries with lower socio-demographic indices (13). Furthermore, the broader societal costs—including strain on educational systems, social services, and the long-term impact on educational and occupational attainment—remain inadequately quantified.

The ideation-to-action framework provides a useful theoretical perspective for understanding the progression from suicidal thoughts to suicidal acts, distinguishing factors associated with the emergence of suicidal ideation from those associated with the transition from ideation to action (14, 15). Within this framework, NSSI has been conceptualized as a behavior that may increase an individual’s capability for suicide through habituation to pain and reduced fear of death. This acquired capability has been proposed as a factor associated with the progression from suicidal thoughts to suicidal behavior; however, these are proposed as potential pathways rather than established causal mechanisms.

Despite the clinical and public health significance of these phenomena, the correlates of NSSI and suicidal behavior remain incompletely understood. Existing evidence is fragmented across psychological, social, and neurobiological domains, and many studies do not clearly distinguish factors associated with the emergence of suicidal ideation from those associated with progression from ideation to suicide attempts. Furthermore, the extent to which the correlates of NSSI are specific to self-injury itself, rather than reflecting comorbid psychopathology or general psychiatric severity, remains an open question.

This narrative review aims to synthesize the available evidence on psychological, social, and neurobiological correlates of the NSSI–suicidal behavior relationship in adolescents. Specifically, we examine cognitive flexibility, family and peer factors, sex differences, hypothalamic-pituitary-thyroid and hypothalamic-pituitary-adrenal axis function, the endogenous opioid system, and dialectical behavior therapy. Throughout this review, we distinguish, where the evidence permits, between factors associated with the emergence of suicidal ideation and those associated with progression from ideation to suicide attempts, as these are increasingly recognized as distinct processes with potentially different underlying mechanisms. This review does not seek to establish causal relationships, but rather to identify consistent patterns, highlight methodological limitations, and suggest directions for future research.

## 2 Methods

### 2.1 Operational definitions of key terms

To ensure conceptual clarity, this review distinguishes between non-suicidal self-injury (NSSI), self-harm, suicidal ideation (SI), suicide attempt (SA), and suicidal behavior (SB). NSSI refers to deliberate, self-inflicted damage to body tissue without suicidal intent. Self-harm is a broader term that may include both non-suicidal and suicidal self-injurious behaviors, depending on the definition used in individual studies. Suicidal ideation (SI) refers to thoughts about engaging in suicidal behavior, ranging from passive wishes of death to active planning. A suicide attempt (SA) is defined as a self-injurious behavior with at least some intent to die. Suicidal behavior (SB) is used as an umbrella term encompassing SI, SA, and suicide death.

Included studies vary in their operationalization of suicidal intent. Some studies explicitly distinguish between NSSI and suicide attempts based on the presence or absence of intent to die, whereas others rely on self-report measures that may not fully capture intent. In synthesizing the literature, we interpret findings according to the definitions provided in each original study and emphasize behavioral outcomes rather than assuming uniform conceptualization across studies.

Beyond the core distinction between suicidal and non-suicidal intent, studies also vary in the frequency or severity thresholds used to define NSSI. Some classify individuals as engaging in NSSI based on a single lifetime occurrence, whereas others require a minimum number of episodes (e.g., five or more) or restrict the definition to behaviors within a specified time frame (e.g., the past 12 months). These methodological differences can substantially influence prevalence estimates and may affect the strength of observed associations between NSSI and suicidal outcomes. For instance, studies using more stringent frequency criteria may identify a more severely affected subgroup, potentially yielding different patterns of correlates than studies using broader inclusion criteria. Where relevant, we note the specific NSSI criteria used in individual studies and encourage readers to consider these operational differences when interpreting the synthesized findings.

### 2.2 Assessment of suicidal intent in the reviewed studies

The studies reviewed vary in their assessment of suicidal intent. Some employed validated self-report instruments, such as the Beck Scale for Suicide Ideation (BSS) or the Self-Injurious Thoughts and Behaviors Interview (SITBI), which directly inquire about the presence and degree of intent to die. Others used structured or semi-structured clinical interviews to distinguish suicide attempts from NSSI based on the reported motivation for the behavior. However, a number of studies, particularly those using large-scale school-based surveys or secondary data analyses, relied on brief self-report items (e.g., “Have you ever tried to kill yourself?”) that may not fully capture the nuanced distinction between suicidal and non-suicidal self-injury. Additionally, some studies use the broader term “self-harm” without specifying whether behaviors were accompanied by suicidal intent. Throughout this review, we note when studies used specific intent assessments versus broader self-report measures and interpret findings in light of these methodological differences. When the assessment of intent is unclear or absent, we use the terminology adopted by the original study while acknowledging the potential for conceptual overlap.

### 2.3 Literature search strategy

This study is a narrative review aimed at synthesizing current evidence on the association between NSSI and suicidal behavior in adolescents. While adopting a structured approach to literature identification, it does not constitute a systematic review, as it does not include formal quality appraisal or exhaustive, protocol-driven selection.

A literature search was conducted in PubMed, Web of Science, PsycINFO, and China National Knowledge Infrastructure (CNKI) for studies published between January 2010 and June 2025. Two articles formally assigned to a 2026 publication volume but available online ahead of print within the search window were retained. This timeframe was selected to focus on contemporary research reflecting current diagnostic frameworks for NSSI. The search strategy combined terms for NSSI, suicidal behavior, and adolescents, supplemented by concept-driven keywords related to psychological, neurobiological, and social-environmental factors.

### 2.4 Study selection and process

Studies were included if they: (1) involved adolescent samples, (2) examined NSSI, suicidal ideation, or suicide attempts, and (3) investigated relevant psychological, neurobiological, or social correlates. Editorials, commentaries, and studies without adolescent data were excluded.

Two authors independently screened titles and abstracts and subsequently reviewed the full texts of potentially relevant articles. Disagreements were resolved through discussion. Selection was guided by conceptual relevance to the theoretical framework of NSSI and suicidal behavior. Given the narrative nature of this review, a Preferred Reporting Items for Systematic Reviews and Meta-Analyses (PRISMA) flow diagram was not generated.

### 2.5 Quality considerations

Consistent with the methodology of a narrative review, a formal risk-of-bias assessment was not conducted. However, the synthesis and interpretation of evidence were informed by methodological considerations, with greater interpretive weight given to peer-reviewed empirical research, longitudinal studies, and systematic reviews where available.

## 3 Association between NSSI and suicidal behavior

### 3.1 NSSI as a risk marker for suicidal behavior

NSSI and suicidal behavior are distinct but frequently co-occurring phenomena. Although NSSI primarily serves emotion regulation rather than the pursuit of death, they share multiple risk factors and developmental pathways. Longitudinal studies suggest that NSSI may serve as a significant risk marker for subsequent suicidal ideation and suicide attempts. For example, a longitudinal study of 193 Chinese adolescents found that NSSI at baseline was prospectively associated with suicidal ideation one year later (16). In a sample of 218 Chinese adolescents with major depressive disorder (MDD) and NSSI, NSSI frequency was descriptively higher among those with versus without suicide attempts (17). A longitudinal study of referred adolescents with mood disorders found that persistent NSSI—rather than remitted NSSI—was specifically associated with higher suicidal behavior, supporting the continuity between NSSI and suicidality (18). Furthermore, a three-year longitudinal study found that persistent NSSI and persistent suicidal ideation share common risk factors and tend to co-occur over time, suggesting that decreases in NSSI may parallel decreases in suicide risk (18).

### 3.2 NSSI severity indicators

Beyond the mere presence of NSSI, its severity—particularly frequency and number of methods used—has been consistently associated with higher suicide risk. Higher NSSI frequency has been positively correlated with suicide attempt status among adolescents with depression, a pattern also observed in a clinical sample of Chinese adolescents with MDD and NSSI (17). Individuals who engage in multiple methods of NSSI may represent a subgroup at particularly elevated risk. In a predominantly adult sample of individuals with a lifetime history of NSSI, a greater variety of NSSI methods—particularly when combined with higher cognitive flexibility—was associated with an increased likelihood of lifetime suicide attempt (19). While NSSI frequency and method diversity are important risk markers at the group level, they do not provide certainty in predicting outcomes for a specific individual. These indicators should be considered in clinical risk assessment, though they cannot reliably identify which particular adolescent will later attempt suicide.

### 3.3 Ideation-to-action framework and acquired capability

The ideation-to-action framework offers a perspective on how NSSI may be involved in the progression from suicidal thoughts to suicidal acts. One key concept is the acquired capability for suicide, which involves reduced fear of self-injury and increased pain tolerance. Longitudinal evidence in young adults suggests that high pain tolerance may be a risk factor for more severe NSSI engagement (20), and experimental studies in adolescents have demonstrated that individuals with a history of NSSI show higher pain thresholds and tolerance (21). Consistent with this, recent work identifying distinct suicidal motivational profiles among adolescents with NSSI found that specific motivation subtypes (e.g., escape-oriented drivers) were significantly associated with elevated suicide attempt risk (22).

A central contribution of the ideation-to-action framework is its explicit distinction between factors associated with the development of suicidal ideation and those associated with progression from ideation to suicidal action. Within this framework, acquired capability for suicide—including increased pain tolerance and reduced fear of death—is conceptualized primarily as a factor associated with the transition from thinking about suicide to acting on suicidal thoughts, rather than with the initial emergence of suicidal ideation. This distinction is critical because many established risk factors for suicidal ideation are relatively poor predictors of which individuals with ideation will go on to make a suicide attempt. NSSI, through its potential to habituate individuals to pain and fear, may therefore be particularly relevant to understanding this transition rather than the initial development of suicidal thoughts. However, it is essential to emphasize that these are potential pathways, not established causal mechanisms, and many individuals with NSSI never develop suicidal behavior.

## 4 Psychological correlates

### 4.1 Cognitive flexibility

Cognitive flexibility—the ability to shift between mental concepts and adapt to new and unexpected situations—has been implicated in the relationship between psychosocial factors and NSSI among adolescents (23). Given its role in adaptive problem-solving and emotion regulation, impairments in cognitive flexibility may be associated with both NSSI and suicide attempts (24). A multicenter cross-sectional study of 2,109 adolescents (aged 12–18 years) found that cognitive flexibility partially mediated the relationship between social support and NSSI, with notable gender differences: the association between social support and cognitive flexibility was stronger in females than in males (23). However, the nature of the association between cognitive flexibility and suicide risk is not straightforward. A systematic review and meta-analysis of 42 studies (5,946 participants) found a small but significant negative association between cognitive flexibility and suicidal thoughts and behaviors (*r* = −0.132, 95% confidence interval [CI] [−0.202; −0.062]), suggesting that higher cognitive flexibility may act as a protective factor against suicidality, although the effect size remains modest (24).

Furthermore, the relationship between cognitive flexibility and suicidal ideation may depend on contextual factors such as life stress. A longitudinal study of 259 psychiatrically hospitalized adolescents found that chronic stress moderated the association between cognitive inflexibility and suicidal ideation: cognitively inflexible adolescents under high chronic stress were more likely to experience increased suicidal ideation severity, supporting a cognitive inflexibility–stress diathesis model (25). This finding was maintained after adjusting for depressive symptoms and relevant covariates (25). In a study of 11,326 pre-adolescent children from the Adolescent Brain Cognitive Development (ABCD) study, cumulative trauma exposure moderated the relationship between cognitive flexibility and current suicidal ideation: higher cognitive flexibility was associated with lower odds of current suicidal ideation only in children with a single lifetime trauma, but not in those without trauma or with two or more traumas (26). These findings suggest that the protective effect of cognitive flexibility may depend on trauma history and cumulative stress exposure.

The relationship between cognitive flexibility and suicide attempts also appears complex. A study of 100 psychiatric inpatient children and adolescents (mean age 14.39 years) found no significant differences in cognitive flexibility (measured by the Wisconsin Card Sorting Test) between those with and without a history of suicide attempts (27). In a small sub-sample with mood disorders only, those with a history of suicide attempt performed better than those without, challenging the simple assumption that cognitive deficits uniformly are associated with higher suicide attempt risk (27). Furthermore, in a predominantly adult sample of individuals with lifetime NSSI, Park and Ammerman (19) examined the interaction between number of lifetime NSSI methods and cognitive flexibility in predicting suicide attempt history. Their findings revealed a complex pattern: the number of NSSI methods was positively associated with a history of suicide attempt, but only among individuals scoring higher on the “alternative strategies” subscale of the Cognitive Flexibility Inventory—a subscale reflecting the perceived ability to generate multiple alternative solutions to difficult situations (19). Among those with lower scores on this subscale, no significant association was observed (19). This result does not support a simple deficit model in which reduced cognitive flexibility directly increases suicide attempt risk. Rather, it suggests that the role of cognitive flexibility may depend on other individual and contextual factors, and that higher self-perceived ability to generate alternatives may, under certain circumstances, coincide with greater engagement in severe self-injurious behaviors (19).

Several possible explanations may account for this counterintuitive finding. First, individuals with higher cognitive flexibility—particularly those who perceive themselves as skilled at generating alternative solutions—may possess a greater capacity to plan and execute complex behaviors. In the context of severe psychopathology and high distress, this capacity could extend to suicidal acts. This interpretation aligns with the finding by Itzhaky et al. (27) that adolescents with mood disorders and a history of suicide attempts performed better on an objective cognitive flexibility task than those without such history. Second, self-perceived cognitive flexibility, as measured by the Cognitive Flexibility Inventory, may not correspond directly to objectively measured cognitive performance. Individuals who overestimate their coping abilities may be at elevated risk when these strategies prove insufficient in the face of overwhelming emotional distress. Third, a greater variety of NSSI methods may itself reflect a broader tendency toward behavioral experimentation. Among individuals with this tendency, higher cognitive flexibility—in the absence of adequate emotion regulation skills—could facilitate the transition from NSSI to more severe forms of self-injurious behavior. These interpretations remain speculative and warrant direct empirical investigation; however, they underscore the importance of moving beyond simple deficit models when examining the role of cognitive flexibility in the relationship between NSSI and suicidal behavior. Further research is needed to clarify the conditions under which different components of cognitive flexibility may serve as risk or protective factors in this relationship.

## 5 Social and contextual correlates

### 5.1 Sex differences

Female adolescents generally report higher rates of NSSI, SI, and SA compared to males, and the associations between unhealthy behaviors and these outcomes show significant sex differences (28), with the sex gap in NSSI widening during mid-adolescence and narrowing in early adulthood (29). A systematic review and meta-analysis of longitudinal studies confirmed that female adolescents have a higher risk of suicide attempts, whereas males have a higher risk of suicide death (30). A longitudinal study following adolescents over nearly three decades found that males had a suicide rate four times higher than females (31). Furthermore, the correlates of NSSI and SI may differ by sex: physical aggression appears more strongly associated with pure NSSI and SI in females, while anger is more strongly linked to these outcomes in males (32). These patterns suggest that sex-sensitive approaches may be valuable for risk assessment.

### 5.2 Family factors

Family dysfunction, high conflict, and low perceived support are well-documented correlates of both NSSI and suicidal behavior in adolescents (33). A longitudinal study of Chinese adolescents found that maladaptive parenting was prospectively associated with NSSI through internalizing symptoms, with FKBP5 gene variation moderating this pathway (33). In a stressful family environment, individuals may adopt NSSI as a maladaptive coping strategy to regulate negative emotions. Among clinical adolescents with a history of childhood maltreatment, stressful life events and negative affect fully mediate the relationship between maltreatment and NSSI, suggesting that childhood maltreatment may increase NSSI risk by heightening emotional distress in response to subsequent stressors (34). Among adolescents with a history of childhood sexual abuse, perceived family support may moderate the relationship between abuse exposure and NSSI risk (35). Similarly, a longitudinal study found that cumulative family risk prospectively predicted NSSI over time, with effortful control partially mediating this relationship (36). These findings highlight the potential importance of family-involved interventions.

### 5.3 Peer influence

Exposure to a friend’s suicidal behavior or suicide death has been identified as a risk factor for subsequent NSSI and suicidal behavior, possibly through social modeling or contagion processes. A longitudinal study of Chinese adolescents found that peer victimization significantly predicted changes in NSSI over time, with this association being significant only among adolescents with low levels of emotion regulation difficulties (37). A short-term longitudinal study identified three pathways through which interpersonal NSSI exposure may be associated with subsequent suicide risk: a mediating pathway through one’s own NSSI engagement, a direct independent pathway, and an amplifying pathway that strengthens the predictive effect of NSSI engagement (38). These findings underscore the importance of considering peer contexts in both risk assessment and prevention efforts.

## 6 Neurobiological correlates

Research on the neurobiological correlates of NSSI and suicidal behavior has increasingly focused on adolescent-specific samples. Studies in this area vary in their design, sample characteristics, definitions of NSSI and suicide attempt, types of biological material, and timing of sampling. These methodological differences can limit the direct comparability of findings. A summary of key studies discussed in this section is provided in **Table 1**.

**Table 1:** Selected neurobiological studies on NSSI and suicidal behavior in adolescents.

System	Study	Design	Population	Sample size (N)	Age (years)	Diagnosis/Comorbidity	NSSI/SA definition	Biological material	Biomarkers	Main findings	Key limitations
HPT Axis	He et al. (39)	Cross-sectional	Depressed adolescent inpatients with vs. without NSSI	222	13–18	Depressive disorder	NSSI: clinical diagnosis; SA: NR	Serum	TSH, FT3, FT4	NSSI associated with ↑ TSH and ↓ FT4; FT3 no difference	Cross-sectional; no SA comparison; single site
	Zhang et al. (40)	Retrospective	Depressed adolescents with vs. without NSSI	454	13–17	Major depressive disorder	NSSI: clinical diagnosis; SA: NR	Serum	TSH, FT3, FT4, bilirubin, uric acid	TSH associated with NSSI in males (OR=0.499); ↓ bilirubin and uric acid in NSSI	Retrospective; no SA comparison; propensity matching for age only
	Ma et al. (41)	Case-control	Male adolescents with first-episode depression with vs. without NSSI	80	Adolescents	First-episode depressive disorder	NSSI: clinical diagnosis; SA: NR	Serum	T3/T4 ratio, FT3, FT4, TSH, testosterone	↓ T3/T4 ratio, FT3, TSH; ↑ FT4 and testosterone in NSSI group	Male only; small sample; no SA comparison
Endogenous Opioid System	Memik et al. (42)	Case-control	Adolescents with NSSI vs. healthy controls	88 (49 NSSI, 39 HC)	12–18	NR (excluded psychosis, bipolar)	NSSI: clinical diagnosis (DSM-5); SA: excluded recent SA	Plasma	β-endorphin, met-enkephalin	↓ β-endorphin in NSSI; β-EP negatively correlated with depression severity	No SA comparison; comorbidity data limited
	Kao et al. (43)	Longitudinal	Adolescents with NSSI disorder undergoing treatment	53	Adolescents	NSSI disorder; comorbid psychopathology	NSSI: clinical diagnosis; SA: NR	Plasma	β-endorphin	↑ β-endorphin associated with ↓ depressive symptoms; no association with NSSI change	Small sample; no SA assessment; treatment confound
	Van der Venne et al. (21)	Case-control	Adolescent NSSI vs. healthy controls	84 (42 NSSI, 42 HC)	12–18	Depression, anxiety (comorbid)	NSSI: clinical interview (DSM-5); SA: excluded current suicidality	Plasma	β-endorphin	↓ basal β-endorphin in NSSI; ↑ pain threshold	Single time point; no SA comparison
HPA Axis	Van der Venne et al. (44)	Case-control	Female adolescent NSSI vs. healthy controls	209 (164 NSSI, 45 HC)	Adolescents	BPD, depression, ACE	NSSI: clinical interview; SA: NR	Saliva, heart rate	Cortisol, α-amylase, HR, HRV	↑ cortisol response to pain with ↑ NSSI severity; ANS changes independent of psychopathology	Female only; no SA comparison; cross-sectional
	Piarulli et al. (45)	Cross-sectional	Female adolescents with NSSI	NR (estimated ~50)	Adolescents	NR	NSSI: clinical diagnosis; SA: NR	Saliva	Cortisol, DHEA-S	Cortisol correlated with distressing urge (r=0.39) and sensation seeking (r=−0.32)	Small sample; no SA comparison; female only
	Reichl et al. (46)	Longitudinal (2-year)	Help-seeking adolescents with NSSI	51	Adolescents	Various; ACE assessed	NSSI: clinical interview; SA: NR	Saliva, hair	CAR, diurnal slope, hair cortisol	ACE moderated HPA-NSSI course: low ACE + high cortisol → short-term NSSI decline then increase	Small sample; no SA comparison; complex interaction effects
	Wang et al. (47)	Cross-sectional	Adolescent mood disorder patients with NSSI, with vs. without SA	79 (49 SA, 30 non-SA)	13–19	Mood disorders	SA: clinical history with intent; NSSI: clinical diagnosis	Serum	ACTH, cortisol, TSH	Female gender risk factor for SA (OR=2.941); low ACTH associated with recent SA	Cross-sectional; small sample; single site

NSSI, non-suicidal self-injury; SA, suicide attempt; SI, suicidal ideation; HC, healthy controls; BPD, borderline personality disorder; ACE, adverse childhood experiences; FT3, free triiodothyronine; FT4, free thyroxine; TSH, thyroid-stimulating hormone; T3, triiodothyronine; T4, thyroxine; OR, odds ratio; β-EP, β-endorphin; CAR, cortisol awakening response; DHEA-S, dehydroepiandrosterone sulfate; ACTH, adrenocorticotropic hormone; HR, heart rate; HRV, heart rate variability; ANS, autonomic nervous system; NR, not reported; ↑, higher/elevated; ↓, lower/reduced.

### 6.1 Hypothalamic-pituitary-thyroid axis

The HPT axis regulates metabolism via thyroid hormones and exerts profound effects on central nervous system development and function. Recent adolescent-specific studies have examined the relationship between HPT axis function and NSSI. He et al. (39) conducted a cross-sectional study of 222 adolescent inpatients aged 13–18 years with diagnosed depression, finding a 40.5% prevalence of NSSI. NSSI was significantly associated with higher thyroid-stimulating hormone (TSH) levels and lower free thyroxine (FT4) levels, whereas no significant differences were observed in free triiodothyronine (FT3) between the NSSI and non-NSSI groups. Zhang et al. (40) performed a retrospective analysis of 454 hospitalized adolescents aged 13–17 years with major depressive disorder (239 with NSSI, 215 without). After propensity score matching, patients with NSSI showed lower levels of total bilirubin and uric acid. Logistic regression revealed that serum TSH was associated with NSSI in male participants (odds ratio [OR] = 0.499), suggesting that NSSI is more likely related to thyroid function in male depressed adolescents. Ma et al. (41) examined 80 male adolescents with first-episode depressive disorder (40 with NSSI, 40 without). Before treatment, the NSSI group showed a lower triiodothyronine (T3)/thyroxine (T4) ratio, FT3, and TSH levels, and higher FT4 and testosterone levels compared to the non-NSSI group. After 6 weeks of sertraline treatment, FT4 and testosterone levels decreased significantly. Logistic regression identified low TSH and high testosterone as independent risk factors for NSSI in male adolescents with depression. These findings collectively indicate that HPT axis dysregulation—manifested as altered TSH, FT3, and FT4 levels—is associated with NSSI in adolescents with depression, with notable gender differences in the underlying mechanisms.

Several sources of heterogeneity may account for the inconsistencies across HPT axis studies. First, the age range and pubertal stage of participants vary across studies. Thyroid hormone levels fluctuate considerably during adolescent development, and studies that do not stratify by age or pubertal stage may obscure stage-specific associations between HPT axis function and NSSI. For example, the studies reviewed here ranged from 13 to 18 years without consistent consideration of pubertal status (39–41). Second, the presence and severity of comorbid depression—itself known to be associated with HPT axis alterations—may confound the observed associations. While all three studies examined adolescents with depressive disorders, the severity and subtype of depression were not consistently controlled for, making it difficult to determine whether HPT axis alterations are specifically associated with NSSI or reflect underlying depressive pathology. Third, studies differ in whether they assess basal hormone levels at a single time point or dynamic measures such as the TSH response to thyrotropin-releasing hormone (TRH) stimulation; single-point measures may fail to capture the dynamic nature of HPT axis dysregulation. Fourth, most studies in this area have been conducted in Chinese clinical samples (39–41), and regional differences in iodine intake and thyroid function, as well as potential genetic and dietary factors, may limit generalizability to other populations. Fifth, the cross-sectional design of all three available studies precludes any determination of whether HPT axis alterations precede or follow NSSI onset. Future research should address these sources of heterogeneity by employing longitudinal designs, stratifying analyses by age and pubertal stage, systematically assessing depression severity and comorbid psychopathology, and including more ethnically and geographically diverse samples.

### 6.2 Endogenous opioid system

The endogenous opioid system consists of widely distributed neurons that produce three families of opioid peptides: β-endorphin (derived from proopiomelanocortin), the enkephalins (met-enkephalin and leu-enkephalin, derived from proenkephalin), and dynorphins (derived from prodynorphin). These peptides are involved in analgesia, reward, addiction, emotion regulation, and stress response regulation.

Memik et al. (42) measured plasma β-endorphin (β-EP) and met-enkephalin (MENK) levels in 49 adolescents with NSSI and 39 controls. Plasma β-EP levels were significantly lower in adolescents with NSSI than in controls, whereas no significant difference was found in MENK levels. β-EP levels showed a negative correlation with depression severity. Kao et al. (43) conducted a longitudinal study of 53 adolescents with NSSI disorder undergoing specialized treatment, with baseline and one-year follow-up assessments. Higher baseline β-endorphin levels were significantly associated with a decrease in depressive symptoms, but no associations were observed between β-endorphin and changes in NSSI, borderline personality disorder symptoms, or pain sensitivity. Van der Venne et al. (21) similarly found that adolescents with NSSI exhibited reduced pain sensitivity and lower basal β-endorphin levels, supporting the opioid deficiency hypothesis. This hypothesis posits that NSSI serves to upregulate the endogenous opioid system to compensate for an opioid deficiency that may result from chronic childhood stress, trauma, or biological predisposition. However, longitudinal evidence remains limited and does not consistently support the hypothesized association between changes in β-endorphin and changes in NSSI frequency. These findings therefore challenge simple homeostatic models and suggest a more complex role of the endogenous opioid system.

### 6.3 Hypothalamic-pituitary-adrenal axis

The HPA axis, as the core hub of the neuroendocrine stress response, has been examined in relation to both NSSI and suicidal behavior. Van der Venne et al. (44) investigated HPA axis and autonomic nervous system responses to pain in 164 female adolescents with NSSI and 45 healthy controls. Increasing NSSI severity was associated with an increasing cortisol response to pain. After adjusting for comorbid psychopathology, greater NSSI severity remained associated with decreased α-amylase levels, decreased heart rate, and increased heart rate variability following pain. These findings suggest an increased pain-related HPA axis response associated with NSSI severity. Piarulli et al. (45) examined whether cortisol and dehydroepiandrosterone sulfate (DHEA-S) patterns are associated with motivational factors for NSSI in female adolescents. Significant correlations were found between cortisol levels and distressing/upsetting urge (*r* = 0.39) and sensation seeking (*r* = −0.32), as well as between the cortisol/DHEA-S ratio and external emotion regulation (*r* = 0.40) and desire to stop NSSI (*r* = 0.40). Reichl et al. (46) conducted a two-year longitudinal study of 51 help-seeking adolescents engaging in NSSI, examining the impact of adverse childhood experiences (ACE) and HPA axis functioning on the course of NSSI. ACE and HPA axis functioning showed interaction effects in predicting NSSI frequency over time. Adolescents with low ACE severity and either an increased cortisol awakening response (CAR) or a flattened diurnal slope showed a steep decline in NSSI frequency in the first year, followed by a subsequent increase in the second year, suggesting that high diurnal cortisol concentrations in the absence of ACE may be favorable for short-term improvement but carry a risk of allostatic load and subsequent increase in NSSI frequency. In contrast, adolescents with severe ACE may benefit from elevated cortisol concentrations, which may be associated with slower but lasting decreases in NSSI frequency.

With respect to suicidal behavior, Wang et al. (47) conducted a cross-sectional survey of 79 adolescent patients with mood disorders and a history of NSSI, of whom 49 (62.03%) had attempted suicide. Logistic regression analysis identified female gender as a risk factor for SA, and low adrenocorticotropic hormone (ACTH) level as a related factor for recent SA. Notably, this study examined correlates of suicide attempts specifically among adolescents already engaging in NSSI—that is, factors associated with progression to suicidal action within an at-risk group—rather than factors predicting the initial emergence of suicidal ideation.

Several sources of heterogeneity may explain the conflicting findings across HPA axis studies. First, studies differ in whether they assess acute versus chronic suicide risk. Second, the presence of depression, childhood trauma exposure, age, sex, psychotropic medication use, timing of sampling, and inpatient versus outpatient status may all influence HPA axis measures. Third, the specific cortisol measure—whether CAR, diurnal slope, hair cortisol, or reactive response—captures different aspects of HPA axis function and may not be directly comparable. Future research that directly compares HPA axis function in adolescent NSSI patients with and without a history of suicide attempt is needed to disentangle these factors.

## 7 Clinical interventions and implications

### 7.1 Dialectical behavior therapy for NSSI and suicide attempts

Among psychosocial interventions, dialectical behavior therapy (DBT) has the strongest empirical support for reducing both NSSI and suicide attempts in adolescents. In a randomized trial with suicidal youth, DBT was associated with significantly higher rates of suicide attempt remission and recovery compared to supportive therapy, as well as lower relapse rates (48). Notably, suicide attempt outcomes improved more rapidly than NSSI outcomes, and the rates of NSSI remission were lower than those for suicide attempts (48). It is possible that the psychological mechanisms maintaining NSSI and suicide attempts differ and respond differently to DBT (48). A multicenter randomized trial of 173 adolescents at high risk for suicide confirmed that DBT significantly reduced suicide attempts and NSSI, with effects sustained through 12-month follow-up (49). A systematic review and meta-analysis of DBT for adolescent self-harm found a significant moderate effect on self-harm behaviors (standardized mean difference [SMD] = 0.43) and a small but significant effect on suicidal ideation (SMD = 0.21), confirming DBT as an effective intervention for reducing both NSSI and suicide risk in adolescents (50). These findings suggest that DBT is a promising intervention for addressing both behaviors, although NSSI may require longer or more specifically tailored treatment strategies.

### 7.2 Implications for clinical assessment and prevention

The evidence reviewed carries several implications for clinical practice, though all should be interpreted with caution given the largely associational nature of the evidence. First, the consistent association between NSSI and subsequent suicidal ideation and attempts underscores the importance of routine screening for NSSI in adolescents presenting with emotional or behavioral difficulties. Assessment should extend beyond the mere presence of NSSI to include its frequency, number of methods, and functions, as these may inform risk stratification (19). Second, the ideation-to-action framework and the concept of acquired capability suggest that clinicians should evaluate not only the presence and severity of suicidal ideation, but also factors that may increase an individual’s capacity to act on suicidal thoughts—such as habituation to pain and reduced fear of death—a concept central to the interpersonal theory of suicide (15) and supported by empirical evidence from adolescents and young adults (20, 21). This recommendation reflects the growing recognition that factors predicting the onset of suicidal ideation may differ substantially from those predicting the transition from ideation to attempts, and that comprehensive risk assessment should address both stages. Third, the psychosocial correlates discussed, including family conflict and exposure to peer suicidal behavior, point to the potential value of involving families in treatment and addressing interpersonal contexts (38, 51). Finally, given the evidence for DBT, integrating DBT-informed strategies into school-based and community programs may contribute to early intervention (48). However, these clinical implications are tentative, as the reviewed evidence does not establish causal relationships.

## 8 Limitations

This review has several limitations. First, it is a narrative review; no formal methodological quality assessment was conducted, and the review may be subject to selection bias. Second, the majority of included studies employed cross-sectional or case-control designs, which preclude causal inference. NSSI and suicidal behavior likely share common underlying factors such as depression, emotion dysregulation, and trauma, meaning that the observed associations may not be specific to NSSI. Third, definitions and measurement instruments for NSSI and suicidal behavior vary across studies, limiting the comparability of findings.

Fourth, psychiatric comorbidity represents a methodological concern that warrants specific attention. Many of the studies cited in this review included adolescents with depressive disorders, borderline personality features, or a history of suicide attempts, yet findings are frequently attributed to NSSI without adequate consideration of the potential confounding effects of comorbidity. For example, it remains unclear whether HPT axis or endogenous opioid system alterations identified in neurobiological studies reflect NSSI per se, the biological signatures of comorbid depression or borderline pathology, or non-specific markers of overall psychopathology severity. Similarly, the association between cognitive flexibility and suicide attempts may be influenced by the severity of co-occurring mood disorders. Future research should systematically assess and control for psychiatric comorbidity, employing more rigorous designs—such as the inclusion of clinical control groups (i.e., individuals with similar psychopathology but without NSSI)—to isolate correlates specifically attributable to NSSI.

Fifth, neurobiological studies are markedly heterogeneous in terms of biological sample types, sampling timing, and the specific biomarkers examined; different cortisol indices capture distinct aspects of HPA axis function and are not directly interchangeable. Sixth, this review focused on a limited set of variables—cognitive flexibility, specific neuroendocrine systems, and family and peer factors—while other important mechanisms such as emotion dysregulation, hopelessness, impulsivity, and digital media influences were beyond its scope. Seventh, most studies were conducted in Western or Chinese clinical settings, and generalizability to other cultural contexts requires caution. Eighth, many of the studies reviewed here examined NSSI in relation to composite measures of “suicidal behavior” that combine suicidal ideation and suicide attempts into a single outcome, which may obscure stage-specific correlates. Future research should routinely report separate analyses for suicidal ideation and suicide attempts. Finally, although NSSI is a robust risk marker for suicidal ideation and attempts, its predictive utility at the individual level remains limited, and the clinical implications discussed herein require further validation through prospective research.

## 9 Conclusion and future directions

Three cross-cutting themes emerge from the evidence synthesized in this review. First, conditional effects characterize many of the correlates examined. The significance of cognitive flexibility for suicide risk depends on contextual factors such as stress and trauma exposure; the role of HPA axis function in NSSI course is moderated by adverse childhood experiences; and the influence of family and peer factors on NSSI varies by sex and developmental stage. This conditionality challenges simple linear models that posit uniform risk or protective effects and instead calls for research designs and clinical approaches that account for these moderating contexts. Second, the specificity challenge remains unresolved. Many of the psychological, social, and neurobiological correlates reviewed here have been examined primarily in samples with comorbid depression, borderline personality features, or other psychopathology. The extent to which these correlates are specifically attributable to NSSI, rather than reflecting shared underlying pathology or general psychiatric severity, cannot be determined from the available evidence. Third, the ideation-to-action distinction has emerged as critical for understanding the role of NSSI in suicidal behavior. Factors associated with the emergence of suicidal ideation differ, at least in part, from those associated with progression from ideation to suicide attempts. NSSI, through its association with acquired capability for suicide, appears particularly relevant to the latter transition. These three themes—conditional effects, the specificity challenge, and the ideation-to-action distinction—highlight the need for research that moves beyond simple bivariate associations toward multi-method, multi-level investigations capable of capturing the complexity of the NSSI–suicidal behavior relationship in adolescents.

This narrative review highlights that NSSI is a significant risk marker for suicidal ideation and suicide attempts in adolescents. Psychological factors such as cognitive flexibility, social factors including sex differences and family and peer environments, and neurobiological alterations in the HPT, endogenous opioid, and HPA systems have been examined as potential correlates of the NSSI–suicidal behavior link. DBT is a promising intervention for reducing both NSSI and suicidal behavior, although differential treatment responses require further investigation.

Future research should employ prospective, multi-method designs that simultaneously assess psychological, social, and neurobiological factors, while carefully controlling for psychiatric comorbidity. Longitudinal studies are particularly needed to clarify the temporal relationships between NSSI and suicidal outcomes, and to distinguish factors associated with the emergence of suicidal ideation from those associated with progression from ideation to attempts. Ultimately, translating this evidence into school- and community-based prevention programs and establishing comprehensive crisis screening networks will be critical for reducing the burden of adolescent suicidal behavior.
